# **A** **fresh look at** ***Clestobothrium crassiceps*** **(Rudolphi 1819) Lühe, 1899 (Cestoda: Bothriocephalidea) two centuries later: first complete description and novel observations from its type-host in the NW Mediterranean Sea**

**DOI:** 10.1007/s00436-025-08571-4

**Published:** 2025-11-24

**Authors:** Laura Muns-Pujadas, Maria Constenla, Sara Dallarés

**Affiliations:** Departament de Biologia Animal, Biologia Vegetal i Ecologia, Universitat Autònoma de Barcelona, 08193 Campus de la UAB, Cerdanyola del Vallès, Barcelona, Spain

**Keywords:** Cestoda, Bothriocephalidae, Parasites, *Merluccius merluccius*, Taxonomy

## Abstract

*Clestobothrium crassiceps* (Rudolphi, 1819) Lühe, 1899 is the type species of the genus *Clestobothrium* Lühe, 1899, originally described from *Merluccius merluccius* (Linnaeus, 1758) in the Mediterranean Sea. Despite its apparently wide host and geographic ranges, this species remains poorly understood due to the lack of detailed morphological, genetic and epidemiological data. In the present study, newly collected materials of *C. crassiceps* from *M. merluccius* off Barcelona, Spain (NW Mediterranean) were used to provide the first complete description from its type-host and locality. An integrative approach was applied, combining traditional morphological techniques with confocal laser scanning microscopy (CLSM), scanning electron microscopy (SEM), molecular, histological and epidemiological data. Confocal microscopy revealed key diagnostic features of the female reproductive system (*i.e.* vagina, Mehli’s gland, uterus tube), while SEM analysis showed, for the first time, different distribution patterns of microtriches along the scolex and strobila. Histological observations showed the attachment mode of the scolex to intestinal folds, causing mild epithelial alterations such as attenuation of the intestinal epithelium. Prevalence and intensity of infestation with *C. crassiceps* were higher in larger fish, suggesting a role for dietary shifts and potential paratenic hosts in transmission. Phylogenetic analysis based on newly generated 28S and cox1 sequences confirmed the monophyly of the genus *Clestobothrium* and highlighted an intraspecific variation comparable to the genetic divergence observed between congeners *C. splendidum* and *C. cristinae*. Based on these results, a morphological reexamination of paratypes of both species was conducted, proposing *C. cristinae* as a junior synonym of *C. splendidum*.

## Introduction

The cestode genus *Clestobothrium* Lühe, 1899, (Bothriocephalidea: Bothriocephalidae) is mainly found parasitizing Gadiformes bony fishes. *Clestobothrium* species can be morphologically distinguished from those belonging to other bothriocephalid genera by the presence of a sphincter surrounding the anterior aperture of the bothridia (Kuchta et al. 2008). Currently, it includes five valid species, *Clestobothrium crassiceps* (Rudolphi 1819) Lühe, 1899*,* the type species, originally described by Rudolphi (1819) (as *Bothriocephalus crassiceps*) from the European hake *Merluccius merluccius* (Linnaeus, 1758) in the Mediterranean Sea off Naples, Italy; *Clestobothrium neglectum* (Lönnberg 1893) Dronen and Blend 2003, described from *Raniceps raninus* (Linnaeus, 1758) in the Baltic Sea off Sweden (Lönnberg 1893); *Clestobothrium gibsoni* Dronen and Blend 2005, described from *Bathygadus macrops* Goode and Bean, 1885 in the Atlantic off the Gulf of Mexico (Dronen and Blend 2005); and *Clestobothrium splendidum* Gil de Pertierra, Incorvaia and Arredondo, 2011 and *Clestobothrium cristinae* Gil de Pertierra, Incorvaia and Arredondo, 2011, described from *Merluccius australis* (Hutton, 1872) off the Patagonian shelf of Argentina and from *Merluccius hubbsi* Marini, 1933 captured off the same locality and also off San Matías Gulf in Argentina, respectively (Gil de Pertierra et al. 2011).

*Clestobothrium crassiceps* remains a scarcely known species despite apparently being widespread, reported from a wide range of fish hosts. Indeed, it has been reported from different *Merluccius* hosts: the type-host *M. merluccius* (Linnaeus, 1758) in the Mediterranean Sea (Rudolphi 1819), *Merluccius bilinearis* (Mitchill, 1814) off the Canadian and USA coasts (Cooper 1918), *Merluccius albidus* (Mitchill, 1818) off the Canadian coast in the NW Atlantic Ocean (McDonald and Margolis 1995), and *Merluccius productus* (Ayres, 1855) off the USA coast in the NE Pacific Ocean (Wardle 1935) as well as in other teleosts, congeneric hosts: *Dissostichus eleginoides* Smitt, 1898 (Gaevskaja et al. 1990, *Urophycis tenuis* Mitchill, 1814 [as* Urophycis musicki* Cohen and Lavenberg, 1884] (McDonald and Margolis 1995), among others) in the Atlantic Ocean and *Aphos porosus* (Valenciennes, 1837) (Cortés and Muñoz 2009) and *Macruronus magellanicus* Lönnberg, 1907 in the Pacific Ocean (Oliva 2001).

The original description of *C. crassiceps* provided by Rudolphi (1819) was very brief and included no measurements or drawings. Cooper (1918) provided the first complete and detailed morphological description of this parasite up to the present date based on specimens collected from *M. bilinearis* off the NW Atlantic, Canadian and USA coasts. Given the sparsely detailed original description of this parasite provided by Rudolphi (1819) based on specimens collected off Napoli, Italy, Cooper (1918) assumed conspecificity of his materials with *C. crassiceps* from the Mediterranean Sea based on the few morphological available data (Rudolphi 1819; Molin 1861; Diesing 1863; Ariola 1896 and 1900; Parona 1899; Barbagallo and Drago 1903). Since then and to date, a few additional studies have focused on specific morphological features of *C. crassiceps*. For instance, Rees (1958) studied in detail the structure of the scolex of this tapeworm and attempted to explore its attachment mode to the intestine of its host but was unable to draw conclusions due to the lack of specimens fixed in situ attached to the intestinal mucosa. Later, Gil de Pertierra et al. (2011) discussed the presence of an apical disk in the scolex, a feature that had been previously mentioned and illustrated by Cooper (1918) and thereafter confirmed by Miquel et al. (2012) using scanning electron microscopy (SEM) on specimens obtained from the type-host captured off Girona coast (NW Mediterranean). Despite these contributions, a complete morphological, ultrastructural and genetic characterization of *C. crassiceps* from the type-host and type locality (*i.e. M. merluccius* from the Mediterranean Sea) is currently lacking.

Given these considerations, the aim of the present study is to provide, the first complete and detailed morphological redescription including ultrastructural details and genetic characterization of *C. crassiceps* from *M. merluccius* based on newly collected material off Barcelona, Spain, in the Mediterranean Sea. Furthermore, the close interaction of this parasite with its host, its mode of attachment are analysed through a histological assessment and quantitative epidemiological data are provided for the analysed population.

## Materials and methods

### Sample collection

A total of 182 specimens of European hake *Merluccius merluccius* (Linnaeus, 1758) were examined for the present study between 2007 and 2023. Of these, 96 specimens were captured in 2007 on board of oceanographic research vessels. Additionally, 62 specimens were captured in 2019 and 24 specimens in 2022–2023 by commercial fishing vessels from the continental shelf of the Balearic Sea (off Barcelona, Spain) at 50–250 m depth and immediately sacrificed by spinal severance (Chervy 2024).

Some specimens (from 2007) were freshly frozen at −20 ºC for further parasitological examination, others (from 2019) were directly fixed in 10% buffered formalin after performing an abdominal incision for the correct fixation of internal organs and the latest (from 2022–2023) were examined fresh few hours after capture.

Once in the laboratory, fish were measured (standard length, SL), weighed (total weight, TW and eviscerated weight, EW), dissected and all organs were examined under the stereomicroscope for the presence of parasites according to a standardized protocol (Chervy 2024). During the examination of the digestive tract of hakes from 2007 and 2019, tapeworms of *Clestobothrium crassiceps* (Rudolphi 1819) Lühe, 1899 were collected, counted and preserved in 70% ethanol. In the case of hakes from 2022–2023, parasites were killed in warm saline solution and a segment of two specimens was cut off and preserved in 100% molecular grade ethanol for genetic analyses.

### Morphological study

A total of seven cestodes recovered from fresh hakes collected in 2022 were stained with iron acetocarmine, dehydrated through an ethanol series, cleared in clove oil and mounted in Canada balsam (Chervy 2024). Before mounting, the tegument of some pieces of strobila were removed with a scalpel blade to allow a better observation of internal structures (Jones 1990).

Illustrations of mounted specimens were performed with a drawing tube attached to an Olympus BH light microscope (Olympus Corporation, Tokio, Japan) (Faculty of Veterinary Medicine, Universitat Autònoma de Barcelona). Measurements were performed with a stage micrometer and are shown as the range values followed, in parentheses, by the mean, the standard deviation, the number of specimens measured and the number of measurements (the latter only when more than one measurement per specimen was taken). Unless otherwise stated, all measurements are in micrometers (µm).

One of the skinned and stained pieces of strobila was soaked in Canada Balsam and examined through confocal laser scanning microscopy (CLSM) using an Olympus FluoView FV1000 confocal microscope (Servei de Microscòpia i Difracció de Raigs X, Universitat Autònoma de Barcelona).

For scanning electron microscope (SEM) examination, one whole specimen was mounted on a SEM stub using conductive double-sided carbon tape and coated with a 40 nm thickness alloy of gold (60%) and palladium (40%) using an Emitech K550X sputter coater and then observed in a Zeiss Merlin high-resolution scanning electron microscope (Carl Zeiss Microscopy GmbH, Jena, Germany) (Servei de Microscòpia i Difracció de Raigs X, Universitat Autònoma de Barcelona) and digital images were obtained.

Microthrix terminology follows Chervy (2009)

Finally, an individual with the scolex still firmly attached to the intestinal mucosa of one host collected in 2019 was embedded in paraffin, sectioned into 5 µm serial sections and stained with hematoxylin and eosin (H&E) for histological observations (Faculty of Veterinary Medicine, Universitat Autònoma de Barcelona).

Voucher specimens are deposited in the Museum für Naturkunde (Berlin, Germany) (collection “Vermes”, catalogue Entozoa, No. ZMB E.7761), the Helminthological Collection of the Institute of Parasitology, Biology Centre of the Czech Academy of Sciences (České Budějovice, Czech Republic) (IPCAS C-498) and the parasitological collection of the Zoology unit of the Universitat Autònoma de Barcelona (Barcelona, Spain) (Nos. C31-C46).

Three syntypes of *C. crassiceps* (collection “Vermes”, catalogue Entozoa, No. 1807-E) from the Museum für Naturkunde were made available for examination, and high-quality images of several voucher specimens (Nos. USNM1321638-1,321,644, USNM1349881, USNM1369433, USNM1392752) were provided by the Smithsonian Institution (Washington D.C., United States). Additionally, two paratypes of *C. splendidum* (No. C-600) and *C. cristinae* (No. C-601) were borrowed for examination from the Helminthological Collection of the Institute of Parasitology, Biology Centre of the Czech Academy of Sciences.

### Molecular analyses and phylogenetic reconstruction

Total genomic DNA was extracted from the last proglottid of two specimens collected in 2022 using Qiagen™ (Valencia, California, USA) DNeasy® Tissue Kit following manufacturer instructions.

The D1–D3 regions of the nuclear large subunit ribosomal DNA (28S rDNA) and partial fragments of the cytochrome c oxidase 1 (*cox1*) mitochondrial gene were amplified (50 µl total volume) by polymerase chain reaction (PCR) using ExcelTaq™ SMOBIO® PCR Master Mix (SMOBIO Technologies, Hsinchu City, Taiwan) containing 5 × concentrated master mix, a mixture of recombinant Taq DNA polymerase, reaction buffer, MgCl_2_ (2 mM), dNTPs (0.2 mM), and enzyme stabilizer. Amplifications of 28S rDNA were performed using the primers and conditions described in Fyler et al. (2009). In this case, 0.5 µl of each PCR primer, and 2 µl of extracted gDNA were added to each amplification tube. For amplification of *cox1* mtDNA gene, the primers and conditions used were those described in Scholz et al. (2013) adding 1 µl of each PCR primer and 4 µl of extracted gDNA to each amplification tube. PCR amplicons were sequenced directly for both strands using the primers LSU5 and 1200 F in the case of 28S rDNA and PBI-cox1F_seq and PBI-cox1R_seq for *cox1* mtDNA (Faculty of Biosciences, Universitat Autònoma de Barcelona).

Obtained sequences were assembled and edited using BioEdit v7 (Hall 1999) and submitted to GenBank under accession numbers PV258606-PV258607 (28S rDNA), and PV258609-PV258610 (*cox1* mtDNA). Sequence alignments were performed using Muscle implemented in MEGA v6. Cestode species selected for comparative sequences of 28S rDNA and *cox1* mtDNA, devoted to phylogenetic analyses, are shown in Table 1. See details for all selected comparative sequences in Brabec et al. (2015). For both alignment sets, pairwise genetic distance matrices, based on uncorrected p-distance and number of base-pair differences, were generated with MEGA v6.

**Table 1:** List of cestode species included for sequence alignments of 28S rDNA and *cox1* mtDNA (devoted to phylogenetic analysis) with details on host species, locality and GenBank Accession numbers

Cestode species	Host species	Locality	GenBank accession numbers	
			28S rDNA	*cox1* mtDNA
*Clestobothrium crassiceps* (Rudolphi 1819) Lühe, 1899	*Merluccius merluccius*	Great Britain, North Sea	KR780884	KR780786
*Clestobothrium crassiceps* (Rudolphi 1819) Lühe, 1899	*Merluccius merluccius*	Off Barcelona, Mediterranean Sea	PV258606	PV258609
*Clestobothrium crassiceps* (Rudolphi 1819) Lühe, 1899	*Merluccius merluccius*	Off Barcelona, Mediterranean Sea	PV258607	PV258610
*Clestobothrium cristinae* Gil de Pertierra, Incorvaia and Arredondo, 2011	*Merluccius hubbsi*	Off Patagonia, Atlantic Ocean	KR780901	KR780830
*Clestobothrium splendidum* Gil de Pertierra, Incorvaia and Arredondo, 2011	*Merluccius australis*	Off Patagonia, Atlantic Ocean	KR780920	KR780827
*Anantrum* n. sp. Overstreet, 1968	*Synodus myops*	Off Ocean Springs, Atlantic Ocean	-	KR780831
*Bothriocephalus scorpii* (Müller 1776) Cooper, 1917	*Myoxocephalus scorpius*	Great Britain, North Sea	-	KR780788
*Bothriocephalus* cf. *carangis* Yamaguti, 1968	*Uraspis uraspis*	Indonesia, Indic Ocean	-	KR780790
*Marsipometra hastata* Linton, 1898	*Polyodon spathula*	Off Mississippi, Atlantic Ocean	-	KR780777

For the *cox1* mtDNA sequences dataset, Gblocks 0.91b implemented on the Phylogeny.fr site (Dereeper et al. 2008) was used to select blocks of evolutionarily conserved sites. Maximum likelihood (ML) and Bayesian inference (BI) algorithms were used for phylogenetic tree reconstruction after the determination of the best-fit model of nucleotide substitution with jModelTest v2.1.4 (Darriba et al. 2012) using the Akaike Information Criterion (AIC) and the Bayesian Information Criterion (BIC), respectively. The best-fitting model selected for ML algorithm was the TIM1 + G model (nst = 6, rates = gamma, ngammacat = 4) while for BI was HKY + G (nst = 2, rates = equal, ngammacat = 4). ML analyses were performed in PhyML v3.0 (Guindon et al. 2010) with a non-parametric bootstrap of 100 replicates. BI analyses were carried out on the Phylo.org site on the CIPRES Science Gateway v3.3 (Miller et al. 2010). Log likelihoods were estimated over 10,000,000 generations using Markov Chain Monte Carlo searches on two simultaneous runs of four chains, sampling trees every 1000 generations. The first 25% of the sampled trees were discarded as ‘burn-in’ and a consensus topology and nodal support estimated as posterior probability values (Huelsenbeck et al. 2001) were calculated from the remaining trees.

### Data analysis

Infestation parameters such as prevalence (P) and mean intensity (MI) (with the 95% confidence interval (CI)) of the cestode parasite *C. crassiceps* were calculated following Bush et al. (1997) for each fish size established range (< 20 cm and > 20 cm standard length), related to the ontogenetic shift in the feeding behaviour and diet of the host (D’Iglio et al. 2022). General fish condition was assessed through Le Cren’s relative body condition index (Kn = EW/(α × SL^β^)) (Le Cren 1951), where α and β are the slope and the intercept of the weight-length relationship representing the entire dataset of sampled fish.

Fish body size (standard length, SL), Kn and MI were first plotted for visually assessing data distribution and tested for normality and homoscedasticity using the Shapiro–Wilk test and Levene's test, respectively. Fish size effect and Kn were tested for P and MI using Generalized Linear Models (GZM), applying a logistic distribution for P and a negative binomial distribution for MI.

Data analysis was performed on RStudio software 2024.12.0 + 467. Statistical significance was set at 0.05.

## Results

### Genetic results and phylogenetic relationships

Two partial identical 28S rDNA sequences (1213 and 1185 nt in length) and two partial *cox1* mtDNA sequences (526 and 549 nt in length) differing by 0.076% (4 nt) were generated from two specimens of *C. crassiceps* from the Spanish coast, NW Mediterranean Sea.

The two newly generated 28S rDNA sequences of *C. crassiceps* were identical to that of *C. crassiceps* from the North Sea and differed by 0.08% (1 nt) from those of congeneric *C. cristinae* and *C. splendidum,* whose sequences were identical.

**Fig. 1:**
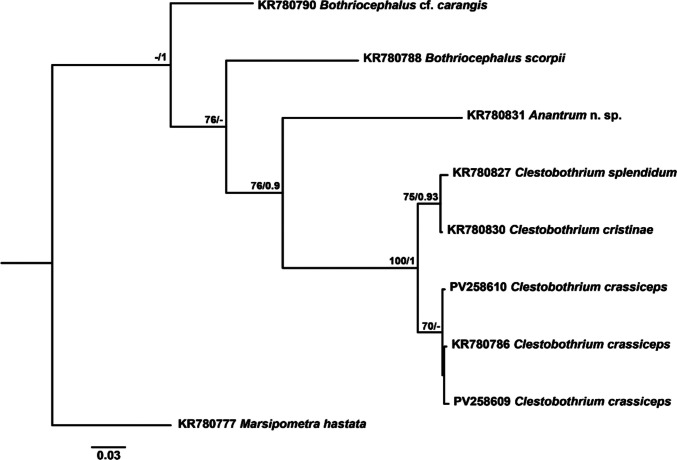
Maximum likelihood (ML) phylogram reconstructed using newly generated *cox*1 mtDNA sequences for *Clestobothrium crassiceps* (PV258609 and PV258610) and retrieved sequences from GenBank for other members. Outgroup: *Marsipometra hastata*. Nodal support from ML and Bayesian inference (BI) analyses indicated as ML/BI. Bootstrap values lower than 70 and posterior probability values lower than 0.9 are omitted. The scale bar indicates the expected number of substitutions per site

A representative phylogenetic tree constructed using *cox1* mtDNA sequences is shown in Fig. 1. The two newly generated sequences of *C. crassiceps* formed a supported clade with the sequence of the same species from the North Sea, from which they differed by 0.57% (3 nt). High bootstrap and posterior probability values characterized the clade comprising *C. cristinae* and *C. splendidum*, which differed by 0.76% (4 nt) between them and by 3.99–4.56% (21–24 nt) and 4.37–4.94% (23–26 nt), respectively, from the newly generated sequences. Overall, all *Clestobothrium* sequences formed a strongly supported monophyletic group clearly differentiated from the other bothriocephalids. With respect to other bothriocephalids included in the analysis, sequences of *Clestobothrium* species differed by 18.06–18.44% (95–97 nt) from *Anantrum* n. sp., by 18.25–18.82% (96–99 nt) from *B. scorpii,* and by 17.68–19.01% (93–100 nt) from *B.* cf. c*arangis.*

### Species delimitation

As can be readily appreciated in the phylogenetic tree constructed from the highly variable *cox1* gene sequences (Fig. 1), *C. splendidum* and *C. cristinae* grouped together forming a highly supported clade, and genetic variation for this gene between these two species is equivalent to that observed between the two newly generated sequences of *C. crassiceps* from the NW Mediterranean. Besides these genetic results, morphological reexamination of paratypes from *C. splendidum* and *C. cristinae* revealed that some features pointed out by Gil de Pertierra et al. (2011) as differential characters to distinguish these two species in their original description should not be considered as such. These authors argued that *C. cristinae* differed from *C. splendidum* in its smaller size and lower number of proglottids, the shape and the length/width ratio of the proglottids and proglottids width/cirrus sac length ratio. These characteristics can vary considerably depending on the worm size either due to allometric growth or intraspecific phenotypic variability. Moreover, Gil de Pertierra et al. (2011) also pointed out as differential characters the absence of genital atrium and testes completely surrounded the posterior region of the ovary in *C. cristinae* and the presence of a vaginal sphincter, together with the equatorial location of the ovary in *C. splendidum*. With respect to the first feature, reexamination of type material revealed the presence of a genital atrium in *C. cristinae*, although less conspicuous than in *C. splendidum* probably due to the smaller size of the worms. The location of the testes relative to ovary was observed to be variable among proglottids but in both species testes partially surrounded the posterior region of the ovary. The presence of a vaginal sphincter in *C. splendidum* was not observed in any of the two species. Vaginal microvilli from the terminal part located posterior to the opening of the cirrus-sac, which were clearly visible, might have been misinterpreted as a muscular sphincter. Finally, a post-equatorial location of the ovary was observed both in *C. splendidum* and in *C. cristinae* during reexamination of types.

Therefore, based on the genetic results and the morphological reexamination of the paratypes from *C. splendidum* and *C. cristinae*, synonymization of both species is proposed herein, with *C. cristinae* becoming a junior synonym of *C. splendidum.*

### Morphological redescription of* Clestobothrium crassiceps (*Rudolphi 1819) Lühe, 1899

*Synonyms: Bothriocephalus crassiceps* Rudolphi 1819; *Bothriocephalus pilula* Leuckart, 1819; *Dibothrium crassiceps* (Rudolphi 1819) Diesing, 1850.

*Type-host:* European hake *Merluccius merluccius* (Linnaeus, 1758) [as *Merluccius vulgaris* Fleming, 1828 sensu Thompson (1844), Molin (1861), Diesing (1863), Ariola (1896 and 1900), Parona (1899) and Barbagallo and Drago (1903)] (Gadiformes: Merlucciidae).

*Type locality:* Mediterranean Sea, off Napoli, Italy.

*Additional hosts and localities:* provided as supplemental information in Table S1.

*Site of infection:* Stomach and intestine.

### Redescription

[Based on five whole mounts of gravid worms, one gravid and two mature (non gravid) worms preserved in ethanol, one piece of skinned gravid strobila devoted to confocal electron microscopy and one mature specimen observed with SEM].

**Fig. 2:**
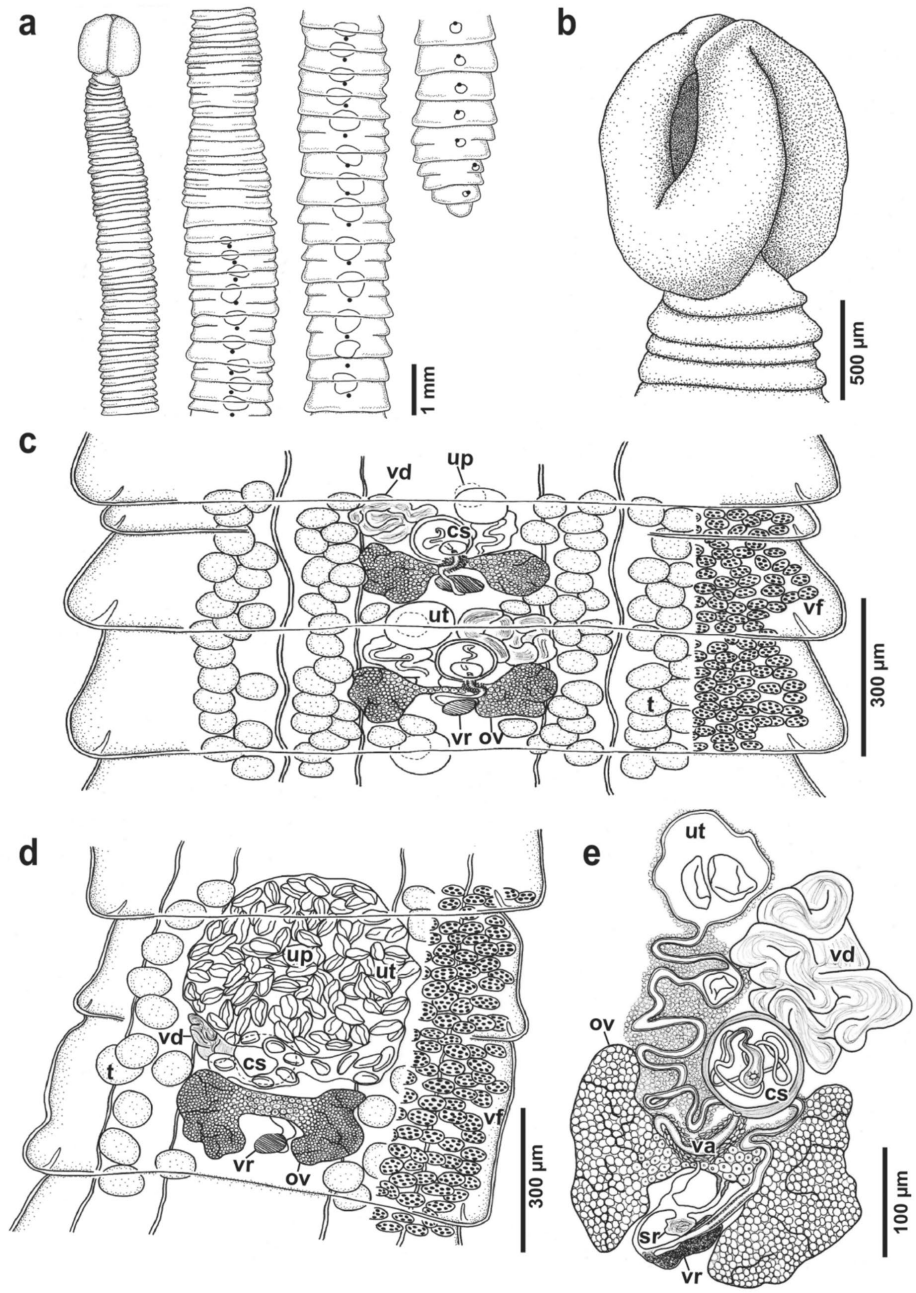
Line drawings of *Clestobothrium crassiceps* (Rudolphi 1819) Lühe, 1899 from *Merluccius merluccius* (Linnaeus, 1758) from the Mediterranean Sea off Barcelona, Spain. **a** outline of entire specimen, dorsoventral view; **b** scolex, dorsoventral view; **c** mature proglottids, dorsal view; **d** gravid proglottid, ventral view; **e** genitalia. Abbreviations: cs, cirrus-sac; ov, ovary; t, testicles; sr, seminal receptacle; up, uterine pore; ut, uterus; va, vagina; vd, vas deferens; vf, vitelline follicles; vr, vitelline reservoir

Cestodes anapolytic, total length 20–34 (24.2 ± 5.8; 5) mm, maximum width 854–1878 (1356 ± 385; 5), at level of late mature-early gravid proglottids (at about three fourths of body length in most specimens) (Fig. 2a). Strobila with 34–122 (60.3 ± 41.4; 5) immature proglottids, 10–20 (15.6 ± 3.8; 5) mature proglottids and 9–37 (25.8 ± 10.5; 5) gravid proglottids (Fig. 2a).

**Fig. 3:**
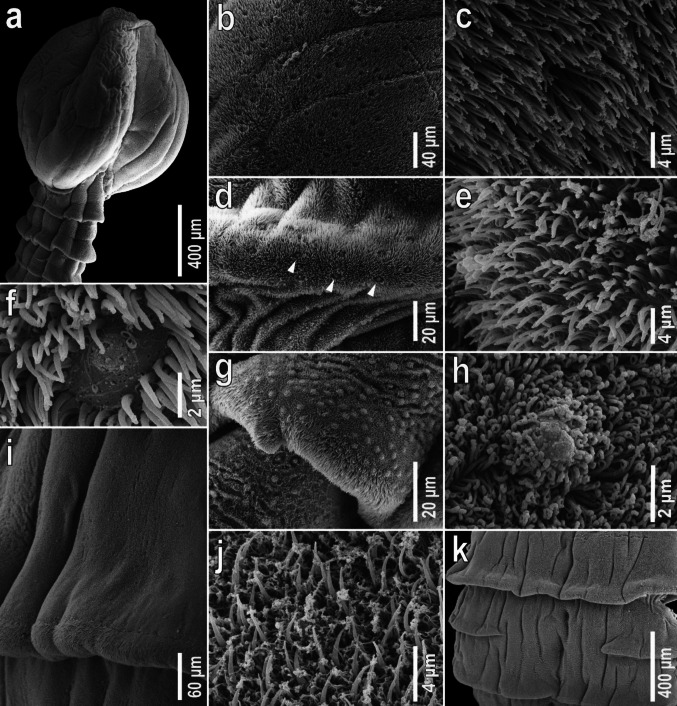
Scanning electron micrographs of *Clestobothrium crassiceps* (Rudolphi 1819) Lühe, 1899 from *Merluccius merluccius* (Linnaeus, 1758) from the Mediterranean Sea off Barcelona, Spain. **a** scolex, dorsoventral view; **b** gladiate spinitriches covering scolex bothridial surface; **c** higher magnification of b; **d** gladiate spinitriches covering the scolex apical surface interrupted by oval worn patches (arrowheads);** e** higher magnification of d; **f** detail of oval worn patches with one papilla-like structure; **g** gladiate spinitriches interspersed with capilliform filitriches covering first immature proglottides; **h** capilliform filitriches with coiled tip, bearing papilla-like structures;** i** capilliform filitriches covering the surface of mature proglottides and gladiate spinitriches covering the velum of proglottides; **j** detail of the gladiate spinitriches covering the velum of proglottides; **k** mature proglottid with spurious segmentation

Scolex acraspedote, compact, subglobular, 592–1238 (980 ± 244; 6) long, 641–1143 (855 ± 195; 7) wide in lateral view, width in dorsoventral view equal to bothridial width; bothridia two in number, tear-shaped in dorsoventral view, with fused posterior margins and very thick and fleshy rims, 534–1202 (894 ± 211; 8) long by 529–686 (585 ± 88; 3) wide (Fig. 2b; Fig. 3a), covered with narrow gladiate spinitriches *ca.* 6.5 long by *ca.* 0.75 wide on their basis (Fig. 3b-c); bothridial aperture tear-shaped, small, opening into a deep and spacious hollow, surrounded by a thick muscular sphincter; proximal bothridial surface with a more or less pronounced medial longitudinal depression continuous with an apical groove that runs along the apex of the scolex between two fleshy lip-like structures (Fig. 2b); scolex apical surface covered with narrow gladiate spinitriches *ca.* 1.7 long by *ca.* 0.45 wide on their basis (Fig. 3d-e); Scolex cover of narrow gladiate spinitriches regularly interrupted by oval worn patches each containing one papilla-like structure, consisting in a circular elevation of the tegument *ca.* 1.5 in diameter (Fig. 3f).

Proglottids craspedote, wider than long, first immature proglottid 108–196 (153 ± 43; 5) long by 534–569 (551 ± 24; 2) wide; first mature proglottid 137–412 (206 ± 116; 5) long by 745–1382 (1059 ± 227; 5) wide (Fig. 2c); first gravid proglottid 244–441 (318 ± 84; 5) long by 863–1634 (1303 ± 301; 5) wide (Fig. 2d); second-to-last gravid proglottid 458–927 (650 ± 199; 5) long by 833–1304 (1144 ± 194; 5) wide, last gravid proglottid narrower than preceding gravid proglottids, 510–1439 (794 ± 370; 5) long by 637–1220 (924 ± 214; 5) wide. First immature proglottids covered with narrow gladiate spinitriches *ca.* 3 long by *ca.* 0.5 wide in their basis interspersed with capilliform filitriches with coiled tip, bearing papilla-like structures consisting in small circular protuberances *ca.* 1.5 in diameter (Fig. 3g-h). Narrow gladiate spinitriches and bumps gradually disappearing in maturing proglottids until only capilliform filitriches and a smooth surface can be observed (Fig. 3i). Velum of all proglottids covered with narrow gladiate spinitriches *ca.* 4 long by *ca.* 0.65 wide on their basis (Fig. 3j). Random presence of spurious segmentation in 14.3–58.8% (33.5 ± 16.9; 5) of mature and 24.3–56.7% (46.8 ± 13.3; 5) of gravid proglottids, dorsoventral margins of spurious segments exceptionally reaching medial line of proglottid (Fig. 2a; Fig. 3k). Boundary between proglottids poorly visible.

**Fig. 4:**
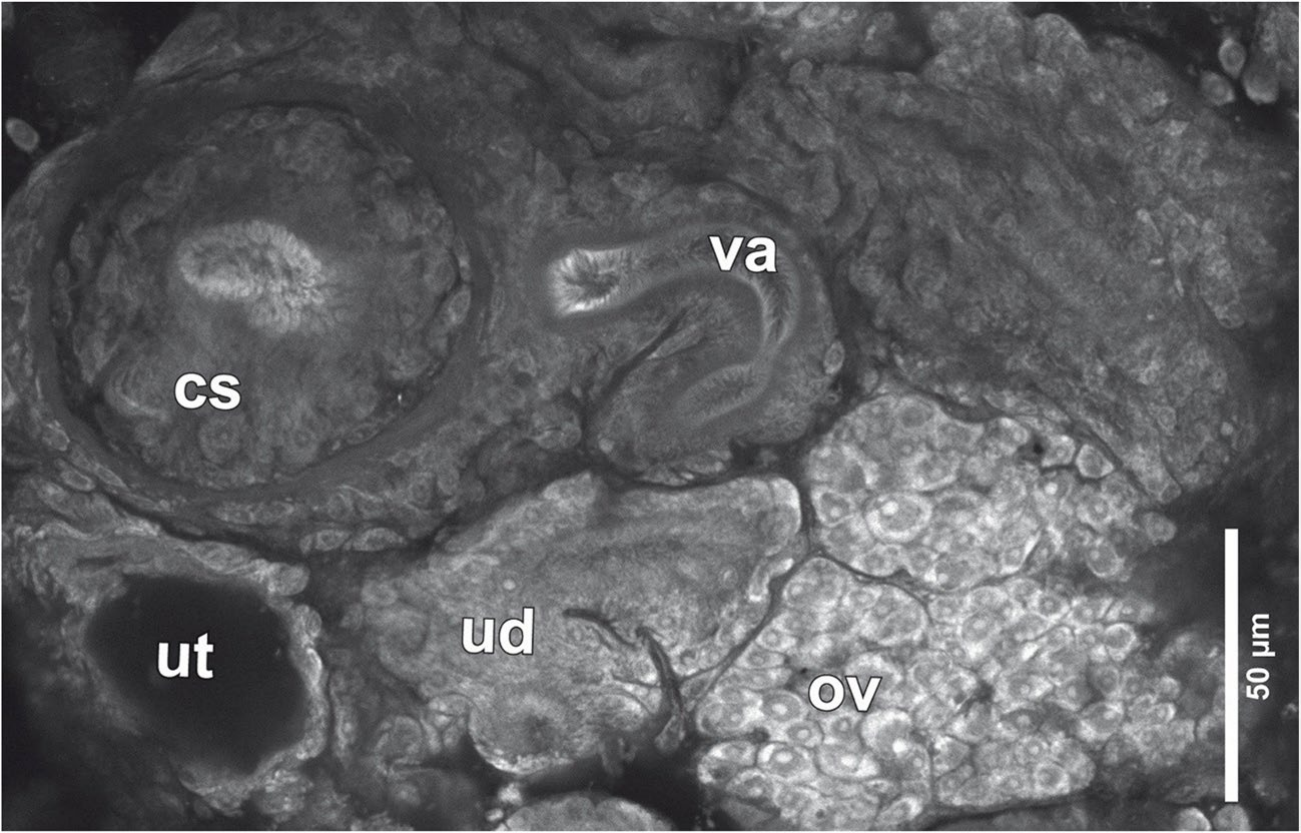
Confocal laser microscopy micrograph showing genitalia of *Clestobothrium crassiceps* (Rudolphi 1819) Lühe, 1899 from *Merluccius merluccius* (Linnaeus, 1758) from the Mediterranean Sea off Barcelona, Spain. *Abbreviations*: cs, cirrus-sac; ov, ovary; ud, uterine duct; ut, uterus; va, vagina

Genital pore dorsal, median, equatorial, immediately posterior to spurious articulations, when present; pore conspicuous, oval, opening into a short hermaphroditic duct or genital atrium (Fig. 2c). Cirrus-sac globular to slightly oval (Fig. 4), vaguely displaced anteriorly with respect to genital pore, irregularly alternating sinistrally or dextrally with respect to genital pore, 81–98 (93 ± 6.2; 6) long by 83–100 (94 ± 5.6; 6) wide, thick-walled (Fig. 2c, e; Fig. 4); cirrus unarmed, coiled inside the cirrus-sac (Fig. 2e). Internal or external seminal vesicle not observed. Vas deferens heavily coiled, compact, located antero-lateral to cirrus-sac (Fig. 2e), almost reaching anterior margin of proglottid (Fig. 2c). Testes slightly oval, 57–88 (67 ± 8.9; 5, 15) long by 43–74 (52 ± 8.5; 5, 15) wide, arranged in a single layer, distributed across the full length of the proglottid and in two lateral fields each divided into an external narrow field and an internal wider field, external and internal lateral fields separated by excretory ducts (Fig. 2c-d); total number of testes per proglottid 30–38 (34 ± 3; 5).

Vaginal opening immediately posterior to opening of cirrus-sac within genital atrium (Fig. 2c). Vagina posterior to cirrus-sac, sinuous, narrowing as it reaches the ovarian isthmus and runs posteriorly between ovarian lobes (Fig. 2e; Fig. 4); slightly widening before joining the oviduct near the posterior margin of ovary (Fig. 2e). Seminal receptacle present, opening into the vagina just at its union with the oviduct (Fig. 2e). Ovary bilobed, H-shaped in dorsoventral view, in posterior region of proglottid, 124–155 (136 ± 12; 5) long by 281–438 (338 ± 63; 5) wide (Fig. 2c-d). Ovarian isthmus ventral, just slightly posterior to cirrus-sac, containing big ova of *ca*. 13 in diameter in fully mature proglottids (Fig. 2c-e). Oocapt small, muscular, located just posterior to ovarian isthmus, opening into a narrow oviduct that widens when joining the vagina and then narrows again as it runs towards the posterior margin of the ovary (Fig. 2e). At this point, it turns anteriorly and receives the vitelline duct from the vitelline reservoir. Vitelline reservoir irregularly oval in shape, ventral to ovary, 26–33 (29 ± 3.6; 3) long by 48–76 (57 ± 17; 3) wide (Fig. 2d-e). Vitelline follicles circummedullar, interrupted dorsally at level of genital pore (Fig. 2c) and ventrally at level of dilated uterus-sac in gravid proglottids (Fig. 2d), oval in shape, 24–48 (37 ± 6.2; 5, 15) long by 19–38 (25 ± 5.3; 5, 15) wide in late mature proglottids, increasing in size in gravid proglottids. Oviduct widening as it runs anteriorly, surrounded by an inconspicuous and diffuse Mehli’s gland located at the level of the vitelline reservoir, and then becoming a uterine tube (Fig. 2e). Uterine tube long, coiled, extending towards the anterior region of the proglottid laterally to cirrus-sac (on the opposite side to vas deferens) (Fig. 2e; Fig. 4). Uterus preformed, tubular, irregularly alternating sinistrally or dextrally with respect to genital pore (Fig. 2c; Fig. 4), enlarged in gravid proglottids forming a circular dilatation full of eggs in anterior region of proglottid (Fig. 2d). Uterine pore ventral, median, near the anterior margin of proglottid (sometimes partially covered by velum of precedent proglottid), conspicuous, round (Fig. 2d).

Eggs oval, 52–67 (59 ± 4.2; 5, 12) long by 36–45 (40 ± 3.3; 5, 12) wide, thin-walled, pale-yellow in colour, operculated (Fig. 2d).

### Remarks

The current redescription based on new materials of *Clestobothrium crassiceps* from *Merluccius merluccius* off the Mediterranean Sea aligns with Rudolphi (1819) original description. However, a precise comparison was not possible due to the absence of measurements and detailed information in the latter. When comparing our materials with those provided by Cooper (1918) redescription of *C. crassiceps* from *M. bilinearis* off the North Atlantic Ocean, some differences mostly related to size variation become apparent. For instance, Cooper measurements of the scolex were on average smaller than those reported herein (592–1238 µm long by 641–1143 µm wide *vs*. 640–1080 µm long by 520–900 µm wide), despite falling within the range observed in present samples. Testes were on average smaller in the new material (67 µm long by 52 µm wide *vs.* 125 µm long by 40 µm wide), and less abundant (30–38 *vs.* 40–50 per proglottid) compared to those provided by Cooper (1918). The cirrus-sac was more globular to slightly oval in the new specimens, whereas it was more elliptical to slightly oval in the Atlantic ones. Its width was similar in both cases measuring 81–98 µm long by 83–100 µm wide in the Mediterranean samples, and 128–162 µm long by 87–116 µm wide in the Atlantic ones (Cooper 1918). Vitelline follicles were also smaller in present samples than in those described by Cooper (1981) (24–48 µm long by 19–38 µm wide *vs.* 60 µm long by 30 µm wide), as well as eggs (52–67 µm long by 36–45 µm wide vs 75 µm long by 40 µm wide), and the ovary was shorter and wider (124–155 µm long by 281–438 µm wide *vs.* 270 µm long by 130 µm wide). In the present redescription the presence of two types of microtriches which Cooper (1918) already noticed and referred to as “cirri” all over the scolex, and “stouter spinilets” on the posterior borders of the proglottids, was confirmed. Therefore, present results confirm the existence of intraspecific size variation between *C. crassiceps* specimens from the Mediterranean Sea and the Atlantic Ocean, as previously suggested by Gil de Pertierra et al. (2011) based on Rudolphi (1819) type material and Rees (1958) voucher specimens from the Atlantic Ocean. In relation to differences observed between *C. crassiceps* and other congeneric species, most of the differences reported by Dronen and Blend (2003) when they redescribed *C. neglectum* and compared it with the description of *C. crassiceps* provided by Cooper (1918) are in agreement with the present redescription of *C. crassiceps* from the NW Mediterranean Sea. However, Dronen and Blend (2003) highlighted that the eggs of *C. neglectum* were smaller and without operculum and that the ovary was larger compared to the observations made by Cooper (1918) of *C. crassiceps*. In the present case though, we observed that the eggs of *C. crassiceps* were actually slightly smaller than those of *C. neglectum* (52–67 [59] long by 36–45 [40] wide *vs.* 65–70 [68] long by 32–40 [35] wide), and that the ovary was larger in *C. crassiceps* compared to *C. neglectum* (338 *vs.* 250 wide). This lack of agreement with Dronen and Blend (2003) comparisons are in all likelihood due to the differences observed between our samples and those provided by Cooper (1918) and highlighted above. Most of the differences indicated by Dronen and Blend (2005) for distinguishing *C. crassiceps* from *C. gibsoni* are consistent with present observations. However, these authors mentioned that the size of the ovary from *C. gibsoni* was wider than the ovary from *C. crassiceps* described by Cooper (1918), but it appeared to be of a similar width compared to present samples (275–400 [340] vs 281–438 [338] wide). This mismatch is, again, likely due to the differences observed between our samples and those made by Cooper (1918). Moreover, *C. crassiceps* specimens from the Mediterranean also display wider than long mature and gravid proglottids, as observed in *C. gibsoni.* This contrasts with Atlantic specimens of *C. crassiceps,* which Cooper (1918) described as having longer than wider mature proglottids.

*Clestobothrium splendidum* (now synonymized with *C. cristinae*, see Sect. 3.2.) differs from *C. crassiceps* in having three pairs of osmoregulatory canals on each side of the proglottids (*vs.* two pairs), and having a straight uterus (*vs.* sinuous) as Gil de Pertierra et al. (2011) already noted. Other differences between both species mentioned by these authors were that *C. crassiceps* did not have a genital atrium, that the ovary was posterior and markedly folliculate, and that testes were not surrounding the ovary posteriorly compared to *C. splendidum*, in which the ovary was equatorial and slightly folliculate, and some testes were surrounding the ovary posteriorly. However, in present samples a conspicuous genital atrium receiving the vagina and the ejaculatory duct was observed, the ovary was post-equatorial in both species and was not folliculate, and also that a few testes were usually located posterior to the ovary in both mature and gravid proglottids. Although Gil de Pertierra et al. (2011) mentioned that the presence of two types of microtriches (*i.e.* capilliform filitriches and gladiate spinitriches) was also a feature shared with *C. crassiceps*, present SEM images also revealed the presence of papilla-like structures similar to those found in *C. splendidum* and referred to as tumuli (dome-shaped evaginations) in Gil de Pertierra et al. (2011) description. The distribution of microtriches between the two species is similar on the surface of proglottids but varies on the scolex; capilliform filitriches were observed only in proglottids from *C. crassiceps*, whereas in *C. splendidum* they were covering the central surface of the apical disk from the scolex.

It is worth to mention an interesting feature of the genus *Clestobothrium*: spurious segmentation. For *C. crassiceps* this feature has been described in some detail and illustrated only by Cooper (1918), while Wagener (1854), Diesing (1863) and Johnstone (1909) only referred succinctly to it. Cooper (1918) considered it as a distinctive feature for *C. crassiceps* and distinguished secondary segmentation occurring in the anterior region of the strobila, which is involved in the formation of new proglottids, from what he called “spurious articulations” (following Wagener’s expression “articulatio spuria”) and are observed in the posterior part of the strobila. He remarked that the dorsoventral margins of these false segments never reached the medial line of the proglottid. Dronen and Blend (2003, 2005) described the same spurious segmentations for *C. neglectum* and *C. gibsoni* using the term “secondary pseudostrobilisation”, while Gil de Pertierra et al. (2011) used indistinctively the terms “secondary segmentation” and “spurious articulation”. As said, Cooper (1918) considered these as different phenomena. As regards the present redescription, spurious articulations were frequently noticed at different degrees of development (*i.e.* from incipient constrictions at lateral margins to well-developed margins crossing all the proglottid and almost reaching the medial line) along the whole strobila. This phenomenon should not be confused with incomplete segmentation, which occurs in several bothriocephalid genera (see Kuchta et al. 2008) and that refers to a partial separation of individual proglottids.

### Histological observations

**Fig. 5:**
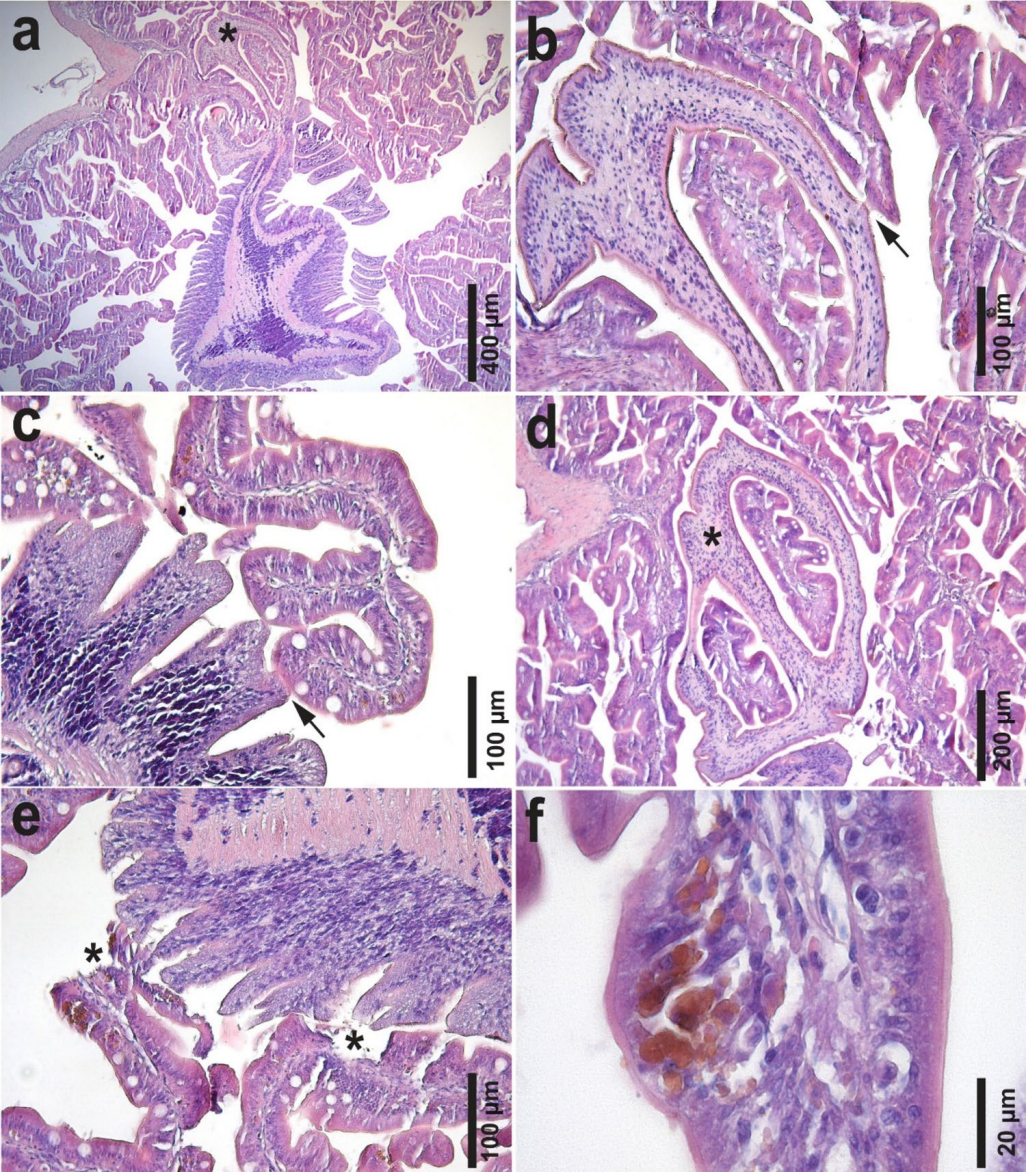
Histological sections (H&E) of a specimen of *Clestobothrium crassiceps* (Rudolphi 1819) Lühe, 1899 attached to the intestinal mucosa of *Merluccius merluccius* (Linnaeus, 1758) from the Mediterranean Sea off Barcelona, Spain. **a** general view of the scolex (*) attached to the intestinal folds; **b** detail of a synechiae between the muscular bothridia and the intestinal mucosa. Note the spinitriches in the tegument in close contact with the adjacent intestinal epithelium (arrow); **c** detail of a synechia (arrow) between the spinitriches of the strobila and the adjacent intestinal epithelium; **d** attenuation of the intestinal epithelium in close contact with the scolex (*); **e** sloughing of the intestinal epithelium (*) in contact with the strobila of the parasite. Note brown-pigmented intracellular debris in the intestinal epithelium; **f** detail of the pigmented intracellular debris

In histological sections, the cestode was observed in the intestinal lumen, attached with the two muscular bothridia to intestinal folds (Fig. 5a). The entire tegument of the scolex was covered with spinitriches (Fig. 5b), which occasionally came into contact with the epithelial surface and developed adhesions or synechiae between them. Some synechiae were also observed between the spinitriches of the strobila and the intestinal epithelium (Fig. 5c). No inflammatory reaction of the fish against the cestode was observed, but there was attenuation (Fig. 5d), mild erosion and sloughing of the intestinal epithelium in contact with the parasite (Fig. 5e). Multifocal, brown-pigmented intracellular debris was also observed in the intestinal epithelium adjacent to the parasite (Fig. 5f).

### Epidemiology

Overall, 41.01% of the examined hakes were infected by *C. crassiceps* with a MI of 1.95 (1.49–2.40 CI 95%) individuals per fish. Differences in parameters of infestation of *C. crassiceps* according to the host size were found. Larger specimens (SL > 20 cm) were significantly more infected than the smaller ones (SL ≤ 20 cm) both in terms of P (47.83% *vs.* 33.72%; GZM, χ^2^= 9.11, p = 0.003) and MI (2.39 (1.65–3.11 CI 95%) *vs*. 1.28 (1.10–1.44 CI 95%) specimens per fish; GZM, χ^2^  = 12.91, p < 0.001). No significant differences were detected between Kn and P or MI of *C. crassiceps* (GLM, p > 0.05).

## Discussion

The present study provides the first complete morphological redescription of *Clestobothrium crassiceps* from its type-host (*i.e*. *Merluccius merluccius*) and type locality (*i.e.* the Mediterranean Sea), after the original description provided by Rudolphi (1819). Given the taxonomic challenges associated with species of *Clestobothrium* and the limited information available on this species, the incorporation of molecular, histological and epidemiological data in this study represents a crucial step for clarifying its diagnostic traits and understanding its potential impact on the fish host.

An integrative approach involving traditional morphological identification and advanced imaging techniques (*i.e*. confocal microscopy, SEM), molecular and ecological data is fundamental for an accurate species characterization (Nadler and De León 2011; Rojas et al. 2025). Morphological features in parasites can be highly variable, not only among developmental stages and host species but also within individuals of the same host and development stage (Kuchta et al. 2024). In this study, confocal microscopy has been proven to be particularly useful in visualizing the distribution of genitalia and female reproductive organs in *C. crassiceps* as some key diagnostic features are challenging to observe with light microscopy. Similarly, it has been also useful for the study of tentacle hook patterns in trypanorhynch cestodes (Elliott 2020; Neitemeier-Duventester et al. 2022).

Phenotypic plasticity can lead to species misidentification, and a good example within the genus *Clestobothrium* is the misinterpretation of *C. cristinae* as a distinct species from *C. splendidum* (Gil de Pertierra et al. 2011), which actually belong to the same species, as demonstrated here. In the present study, molecular results revealed minimal genetic divergence between these two species, comparable to the intraspecific variation observed within specimens of *C. crassiceps* from the same sampling area and fish host. Moreover, morphological reexamination indicated that previously identified distinguishing traits between these species reported by Gil de Pertierra et al. (2011) were not correctly assessed and that some of them fell within the expected range of phenotypic variation, which further justifies the synonymization of both species. A similar pattern of morphological variability was observed in *C. crassiceps*, potentially linked to host-related or geographical variation. Interestingly, molecular data showed that *C. crassiceps* sequences from the Mediterranean were more closely related to those from the North Sea than to each other, suggesting a genetically homogeneous population of this parasite across the Atlantic and Mediterranean. However, additional genetic data from more specimens and geographic locations would be necessary to understand the genetic variability and population structure of this species across its whole distribution range.

The morphology of the scolex and their associated structures (*e.g.,* bothridia, acetabula, rostellums) in cestodes determines their attachment mechanisms and the extent of interaction with host tissues. In *C. crassiceps*, the presence of two bothridia suggests an attachment mode adapted to grasping the intestinal folds, although the potential effects on host tissue remain unknown. To date, only Rees (1958) suggested that these bothridia might function similarly to the acetabula in Cyclophyllidea, generating suction and possibly engulfing portions of the intestinal mucosa through the sphincter contraction. However, this hypothesis could not be confirmed to date due to the lack of specimens fixed in situ attached to the intestinal mucosa. The present study provides for the first-time a histological section of the scolex of *C. crassiceps* attached to the intestinal mucosa of its type-host, *M. merluccius*, where the engulfing of intestinal folds by the bothridia is confirmed. Although cestodes generally do not cause severe damage to the intestinal wall because they do not penetrate deeply into it (Bosi et al. 2022), the morphology of the scolex and its anchoring structures are responsible for the extent of the damage they can cause to the host (Scholz et al. 2021). The comparison of our findings with those on other Bothriocephalid species highlights both similarities and differences in attachment mechanisms and their associated damage in fish hosts. For example, the Asian fish tapeworm (*Bothriocephalus acheilognathi* Yamaguti, 1934) attaches to the gut wall by its bothria, which engulf the intestinal folds, causing compression of the mucosal epithelium, focal pressure necrosis, haemorrhage, ulceration and focal inflammatory response in cyprinids (Scholz et al. 2012). Although a similar attachment is observed by *C. crassiceps* in the hake’s intestine, no significant alterations of the intestinal mucosa in contact with the scolex are observed (neither necrosis, haemorrhages, nor inflammation). The bothridia of *B. scorpii* (Müller, 1776) Cooper, 1917, were reported to function as elongated pincers, gripping the folds of the intestinal mucosa from its hosts *Scophthalmus maximus* (Linnaeus, 1758) and *Rhombus laevis* (Linnaeus, 1758), but this interaction resulted in minor structural changes at the attachment site including reduced epithelial thickness and a slight increase of fibrous tissue (Rees 1958; Davey and Peachey 1968). A more pronounced response caused by *B. scorpii* was observed in *Pseudophycis bacchus* (Forster, 1801)*.* The host responded by depositing collagenous connective tissue around the scolex, leading to villi loss at the attachment area, marked fibrosis, and scolex encapsulation forming a swelling in the fish gut wall (McKinnon and Featherston 1982). Mild histological alterations (synechiae, attenuation of the intestinal epithelium with intracellular waste material and scarce erosion of the intestinal epithelium) have been found associated to the presence of *C. crassiceps* in the intestinal lumen. Other authors also reported reduction of epithelial thickness associated to the presence of bothriocephalids in the intestine (Rees 1958; Davey and Peachey 1968; Scholz et al. 2012). These changes together would indicate a functional impairment of the intestine, although very mild in the case of *C. crassiceps*. Changes in enzymatic activity and protein synthesis, or even secretion of toxic material is also confirmed in other bothriocephalids (Scholz et al. 2021). Heavy infections by cestodes of this group in other fish species such as *Eubothrium salvelini* (Schrank, 1790) Nybelin, 1922 in coho salmon *Oncorhynchus kisutch* (Walbum, 1792), have been shown to negatively affect growth, condition and fitness or even leading to fish mortalities (*e.g., Triaenophorus nodulosus* (Pallas, 1781) Rudolphi, 1793 in juvenile salmonids) (see Scholz et al. 2021). However, no significant correlations were detected between hosts’ condition factor and parasitological indicators in the present case, indicating that infections by *C. crassiceps* do not compromise hake condition.

Basic aspects of the biology of *C. crassiceps*, such as their life cycle, remain also poorly understood. Though, it can be presumed what has been described for other bothriocephalidean species: planktonic crustaceans (*i.e*. copepods), where procercoid and plerocercoid larvae develop, may act as first intermediate hosts, and fish species are the definitive hosts, becoming directly infected after consuming the former (Kuchta and Scholz 2007; Scholz et al. 2021). In some cases, a paratenic host (*i.e*. a fish) is also involved, as it has been reported in *Bothriocephalus gregarius* Renaud Gabrion and Pasteur, 1983 (Morand et al. 1995). In *B. acheilognathi* and *Bothriocephalus pearsei* Scholz, Vargas and Moravec, 1996, it was shown that the transmission of adult parasites can occur from fish to fish through predation by infected host, a phenomenon known as postcyclic transmission (Hansen et al. 2007; Scholz et al. 2012). In the present study, fish size from the definitive host of *C. crassiceps*, *M. merluccius,* appeared to be an important contributing factor influencing *C. crassiceps* abundance, suggesting that host feeding rates and/or dietary shifts are important keys of parasite transmission. Indeed, *M. merluccius* undergoes dietary shifts due to ontogenetic shifts in feeding behaviour according to body size. Juvenile hakes (< 15 cm total length) exhibit an opportunistic feeding strategy consuming euphausiids, mysids and decapod crustaceans (Modica et al. 2011; D’Iglio et al. 2022), some of which may act as first intermediate hosts for *C. crassiceps* larvae. As they grow (15–20 cm total length), their diet become more selective dominated by small fishes and suprabenthic crustaceans (Carrassón et al. 2019; D’Iglio et al. 2022), and finally mature hakes (> 20 cm total length) adopt a piscivorous diet (D’Iglio et al. 2022). This transition to a more piscivorous diet increases the chances of ingesting infected prey, which could act as paratenic hosts (*i.e*. fish) for *C. crassiceps*, leading to a higher parasite load in larger individuals, as present findings indicate. This trend was also observed in *Bothriocephalus barbatus* Renaud Gabrion and Pasteur, 1983 and *B. gregarius,* in which dietary shifts towards a piscivore diet increased the probability of parasitation while maintaining copepod ingestion as an additional infection route (Robert et al. 1990).

Beyond transmission dynamics, understanding the host range of the cestode *C. crassiceps* is essential to clarify its ecological and evolutionary relationships within the Bothriocephalidea. Bothriocephalideans tapeworms generally exhibit strict host specificity, with about 90% being stenoxenous or oioxenous (infecting one or two host species), while the remaining are euryxenous, infecting a wide range of different host species (Kuchta and Scholz 2007). *Clestobothrium crassiceps* and several species from the genus *Bothriocephalus* have been found in up to 30 marine fish species, Gadiformes, Scombriformes, Pleuronectiformes and Lophiiformes, amongst others (Cooper 1918; Schmidt 1986). Moreover, *Clestobothrium* species have been recently reported from latest fish hosts (*e.g., C. neglectum* in *Raniceps ranius*). Thus, some hosts records previously attributed to *C. crassiceps* could correspond to other congeners. The host specificity of many marine taxa remains unclear due to unresolved taxonomy and limited definitive host data (Kuchta and Scholz 2007). In particular, the taxonomic status of several *Merluccius* species which host *C. crassiceps* has remained uncertain for decades, especially from closely related species from the Pacific Ocean (*i.e. Merluccius productus, M. angustimanus* Garman, 1899 and *M. hernandezi* Mathews, 1985) that exhibit overlapping morphological traits (Lloris et al. 2005; Silva-Segundo et al. 2011). A recent molecular study suggested that rare *Merluccius* morphotypes from the South Pacific and South Atlantic oceans, previously suspected to be cryptic species, are the result of recurrent hybridization events between *M. gayi* Guichenot, 1848 and *M. australis* or *M. hubbsi* (Pérez et al. 2021). Such taxonomic misidentifications could influence host records for *C. crassiceps* and affect our understanding of its host specificity. Altogether, these taxonomic uncertainties and host range ambiguities highlight the need for integrative taxonomic approaches to accurately assess the host specificity of *C. crassiceps* and its congeners.

## Conclusions

The present study provides the first complete redescription of *C. crassiceps* from its type-host and type-locality. We highlight the importance of integrative taxonomy combining traditional morphology with advanced imagining techniques and molecular data to avoid species misidentification due to phenotypic plasticity commonly observed in *Clestobothrium* species. Indeed, despite intraspecific size differences observed between *C. crassiceps* specimens from the Atlantic and Mediterranean, molecular data revealed a genetically homogeneous population of this parasite across both regions. Moreover, genetic results and morphological reexamination supported the synonymization of *C. cristinae* with *C. splendidum,* previously described as distinct species from *M. australis* and *M. hubbsi* off the Patagonian shelf of Argentina. Histology of the *C. crassiceps* specimen attached to the intestinal mucosa of *M. merluccius* provided new insights into the interaction between the attachment structures and host tissue, showing only mild effects. Further studies using an integrative approach as the present study should be more widely applied in parasite taxonomy and ecology across different taxa and regions, to improve our understanding of host-parasite interactions, species boundaries and host specificity in marine ecosystems.

## Data Availability

Data will be made available upon request. Molecular sequence data are available in GenBank under accession numbers PV258606-PV258607 (28S rDNA), and PV258609-PV258610 (*cox1* mtDNA). Voucher specimens are deposited in the Museum für Naturkunde (Berlin, Germany) (collection “Vermes”, catalogue Entozoa, No. ZMB E.7761), the Helminthological Collection of the Institute of Parasitology, Biology Centre of the Czech Academy of Sciences (České Budějovice, Czeck Republic) (IPCAS C-498) and the parasitological collection of the Zoology unit of the Universitat Autònoma de Barcelona (Barcelona, Spain) (Nos. C31-C46).
